# A case of synovial sarcoma of the right mid-thigh and literature review

**DOI:** 10.3389/or.2024.1445143

**Published:** 2024-12-20

**Authors:** Jing Zhang, Zhengyi Li, Guoqiang Guo, Chunchun Jin, Meifang Deng

**Affiliations:** Department of Ultrasound, The First Affiliated Hospital of Shenzhen University, Shenzhen Second People’s Hospital, Shenzhen, China

**Keywords:** synovial sarcoma, mesenchymal neoplasm, biphasic, ultrasound, literature review

## Abstract

Synovial sarcoma (SS) is a rare and malignant mesenchymal neoplasm. We report a case of a 16-year-old Chinese female diagnosed with biphasic synovial sarcoma. The imaging features, surgical procedures and pathological results of the lesion were described in detail. Additionally, we conducted a review of the literature on synovial sarcoma of the thigh over the past 2 decades, identifying a total of 25 relevant case reports and summarizing the characteristics of these cases. Synovial sarcoma has a high degree of malignancy, with a high recurrence and metastasis rate, and a 5-year survival rate of 36%–76% and a 10-year survival rate of 20%–63%, so early detection of the lesion and preoperative differential diagnosis are of paramount importance in the treatment of patients.

## Introduction

Synovial Sarcoma (SS) is a highly aggressive malignant tumor derived from undifferentiated mesenchymal cells, which accounts for 5%–10% of malignant tumors of mesenchymal tissue (1). Synovial sarcoma can occur in all age groups, but predominantly in young adults. The age of patients is generally between 20 and 40 years old, and there is no significant difference between men and women. It can be found in various parts of the body, preferably in the vicinity of large joints, and is closely related to tendons, tendon sheaths, synovial bursae, joint capsules. It can also occur in parts without synovium, and usually does not invade the joint itself (2). SS is not differentiated from synovium but consists of spindle cells of unknown origin and/or nests of epithelioid cells of varying degrees of differentiation. According to the presence of both types of cells, SS could be divided into monophadiform and biphasic types, or an undifferentiated type containing only poorly differentiated fibrous spindle cells (2). No known risk factors for synovial sarcoma have been identified, but the disease is associated with the t (X; 18) (p11; q11) chromosomal translocation. SS18(SYT)-SSX fusion gene testing is the gold standard for the diagnosis of SS, while immunohistochemistry and imaging tests are used as auxiliary diagnostic tools (3). SS has a poor prognosis, with reported 5-year survival rates of 36%–76% and 10-year survival rates of 20%–63%. More than 50% of patients recurring within 2 years, while about 40% of patients will metastasise to the lung, bone, and other sites (4). Surgery supplemented by postoperative radiotherapy/chemotherapy is considered the mainstay of treatment.

We report on a 16-year-old Chinese female diagnosed with synovial sarcoma who underwent two surgical treatments and regular chemotherapy. Follow-up did not reveal any recurrence of the original disease, but bilateral lung metastases were detected. We also reviewed the relevant literature on synovial sarcoma of the thigh over a 2 decades period.

## Case presentation

A 20-year-old female presented to the hospital with a history of post-exercise pain for more than 11 months. She found a mass on her right thigh that was painful and swollen after compression.

Physical examination: palpable mass on the right medial thigh was poorly demarcated from the surrounding tissues on palpation, with pressure pain accompanied by swelling, and the skin around the mass was normal in color and inactive.

**FIGURE 1:**
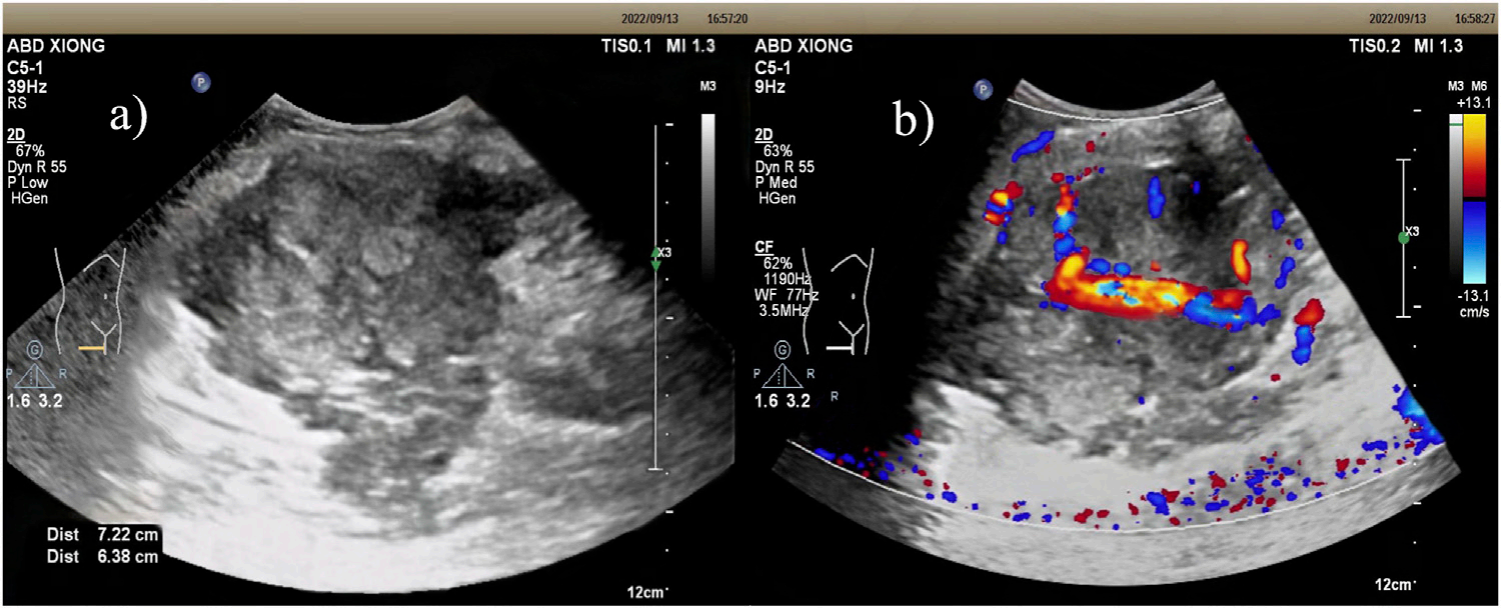
Ultrasound: **(A)** Slightly hypoechoic mass within the vastus medialis muscle of the right middle and lower thigh, with irregular morphology, poorly defined borders, and poorly homogeneous internal echogenicity. **(B)** Color Doppler: richer blood flow signals were seen within the mass.

**FIGURE 2:**
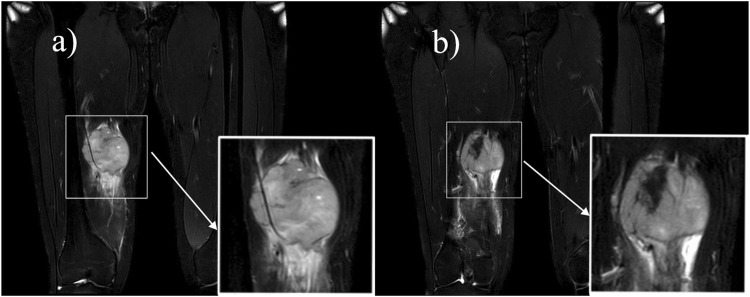
MRI: A soft-tissue mass of the medial muscle of the right mid-thigh, which was lobulated and showed a “Triple-sign” of mixed high and low signals on T2-weighted images. **(A)** Speckled high signal is seen within the lesion, suggesting a localized cystic lesion, and the lesion encircles the superficial femoral artery. **(B)** Fibrous components and old hemorrhages in the lesion show low signal.

**FIGURE 3:**
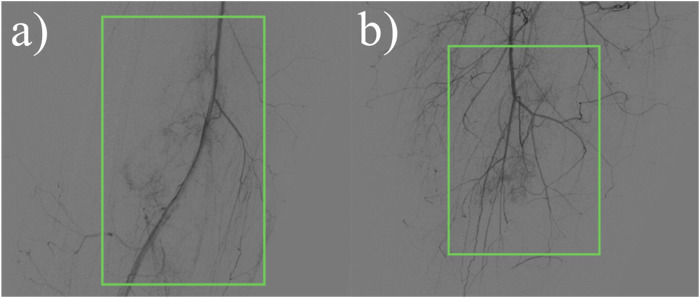
X-ray radiographic contrast:Irregular more massive tumor vascular shadows are seen in the soft tissue area of the right mid-femoral femur, supplied by the right superficial femoral artery and branches of the deep femoral artery, and dense tumor staining is seen in the parenchymal stage. **(A)** Front view. **(B)** Side view.

**FIGURE 4:**
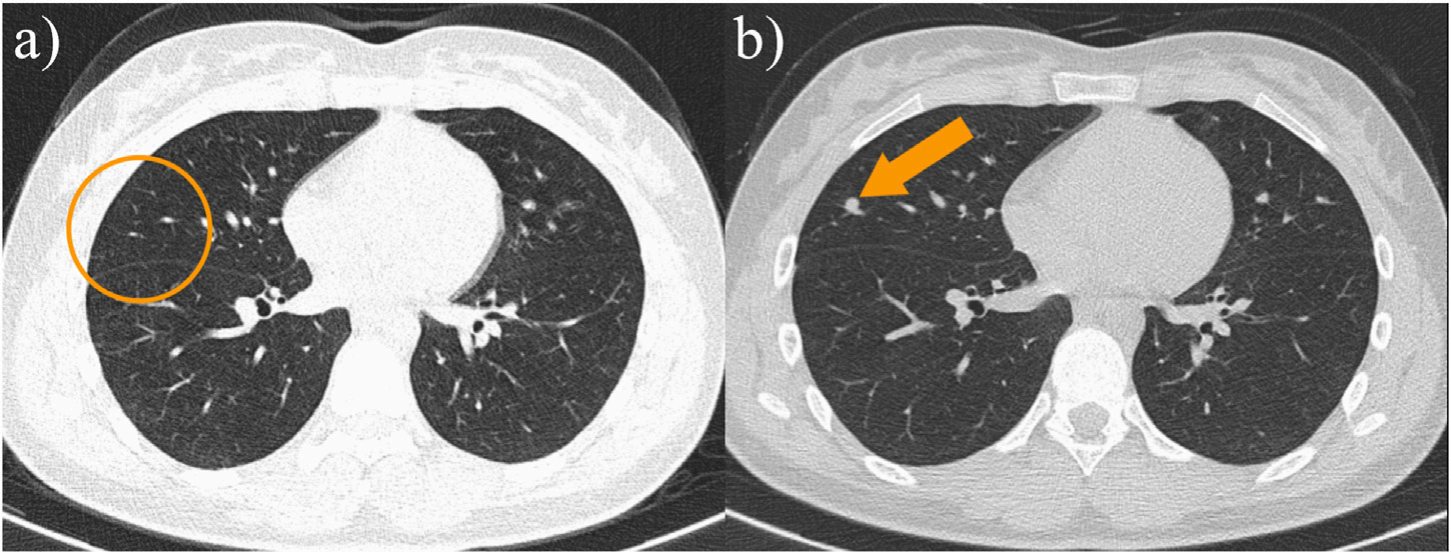
**(A)** The patient’s chest CT at the time of initial diagnosis did not reveal a definite metastatic lesion; **(B)** The patient’s follow-up chest CT 13 months after surgery showed the appearance of a new paravascular soft tissue nodule, and lung metastasis was considered.

Ultrasound: A slightly hypoechoic mass measuring about 7.2 × 5.6 × 6.4 cm was found in the medialis longus muscle of the lower middle thigh on the right side, with an irregular shape, poorly defined borders, and poorly homogeneous internal echogenicity, and the peripheral superficial femoral vein and saphenous vein of the mass were poorly displayed (Figure 1A), and color Doppler showed that rich blood flow signals could be seen in the mass (Figure 1B). Ultrasound suggests that further examination is recommended. MRI:Soft tissue mass in the medial muscle of the middle right thigh (short and long adductors in the center, and part of the medial femoral muscle), foliated, with slightly high signal in T1WI, high signal in T2WI, and widely diffusion-restricted in DWI sequences; the mass was clearly delimited from the right femur, with intact cortex, and no abnormal signal foci were seen in the bone marrow cavity (Figure 2). The MRI shows a high likelihood of malignancy and an enhanced scan is recommended. X-ray radiography showed irregular and massive tumor vascular shadow in the soft tissue area of the right mid-femoral femur, which was supplied by the branch of right superficial femoral artery and deep femoral artery, and dense tumor staining was seen in the parenchymal stage, and there was no obvious venous early appearance (Figure 3). X-ray radiography showed that a soft tissue malignant tumor was considered in the middle of the right femur. Chest CT showed no significant metastases in both lungs (Figure 4A).

**FIGURE 5:**
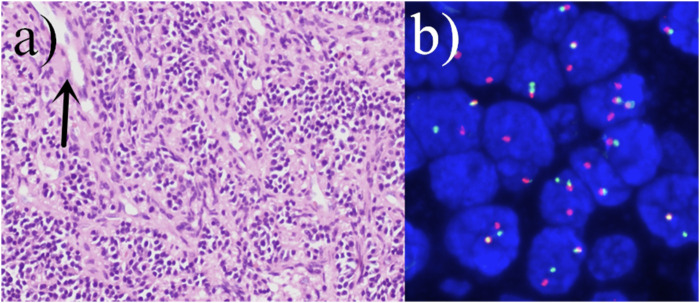
**(A)** The tumor cells are densely proliferated, with flaky, nested mass, striated distribution, infiltrative growth, the nuclei are ovoid, fat pike-shaped, with visible nucleoli, and the nuclear schizophrenia is easy to be seen (hematoxylin and eosin staining; magnification, ×400). Visible characteristic cleft-like space (arrow). **(B)** Positive fluorescence *in situ* hybridization (FISH) analysis of the SYT (SS18) gene rearrangement.

The patient underwent surgery. The operation showed that the tumor was wrapped in the sarcolemma, without complete capsule, and was friable, partially fish-like, wrapping the femoral artery and saphenous nerve. The boundary between the tumor and the surrounding tissue was not clear, and it extended to the deep tissue. Because the tumor was too large and poorly demarcated from the surrounding tissues to be completely resected, local tumor tissue was excised and sent for pathological examination. Microscopic manifestation: the tumor cells are densely proliferated, with flaky, nested mass, striated distribution, infiltrative growth, the nuclei are ovoid, fat pike-shaped, with visible nucleoli, and the nuclear schizophrenia is easy to be seen (Figure 5A). Immunohistochemistry: CD99 (partially+), Bcl-2 (partially+), EMA (mostly+), S100 (−), Desmin (−). Molecular pathology:SS18 gene translocation testing showed that 55 cells exhibited separated red and green signals among 100 counted cells, accounting for 55%. The reference value for a positive result is >15%. Therefore, the result is considered positive, indicating the presence of SS18 gene translocation (Figure 5B). The combination of immunohistochemical and molecular test results was consistent with synovial sarcoma. Two postoperative chemotherapy sessions were performed, with a chemotherapy regimen of biliverdin, cisplatin, and isocyclophosphamide. The second operation was performed 2 months after the operation. The operation showed that the tumor was solid, located below the deep fascia, the tumor was large with unclear borders, and the proximal part of the tumor was obviously adherent to the saphenous vein and femoral artery, encircling the vascular bundles and femoral nerve. The tumor invades the suture muscle superficially, the medial retractor muscle group deeply, and part of the medial femoral muscle distally. Regular chemotherapy was administered postoperatively, and the treatment regimen was liposomal isocyclophosphamide and doxorubicin.

Postoperative follow-up of the patient showed no definite recurrence in the right lower extremity.13 months after surgery, chest CT showed multiple metastases in both lungs (Figure 4B). The patient is now on regular chemotherapy with routine follow-up.

## Discussion

Synovial sarcoma is a rare, malignant, highly graded soft tissue tumor. The age-adjusted incidence of this subtype is 00.81 per 1 million children and 1.42 per 1 million adults (5). We searched the literature for the past 2 decades and found a total of 25 reports of cases of synovial sarcoma of the thigh (1, 6–29) (Table 1). These cases involved patients ranging in age from 13 to 75 years with a mean age of 38.2 years. There were 14 male patients (56.0%) and 11 female patients (44.0%). A total of 25 lesions were involved in these 25 reports in the literature, 14 of which were located on the left side and 11 on the right side. The size of the lesions was documented in 20 reports showing that the maximum diameter of all lesions was more than 3 cm, and the maximum diameter of 10 lesions was more than 10 cm, accounting for 50%. In our patient, the maximum diameter of the lesion was 7.2 cm. These reports indicated that the size of the lesions was generally large, which may be related to the fact that the clinical manifestations of the disease were not obvious in the early stage and did not attract much attention from the patients. As a result, by the time the patient is seen, the lesion is already relatively large.

**TABLE 1:** Reported cases of primary synovial sarcoma of the thigh.

Case no.	Year	Author	Gender	Age	Site	Size (cm)	Pathologic classification	Recurrence	Metastasis	Outcome	Molecular pathology	Immunohistochemistry (IHC)	Supply artery	Infiltration
	2023	Our case	F	20	The middle and lower part of the right thigh	7.2 × 5.6×6.4	Biphasic	N	Y	N	SS18	CD99 (+),Bcl-2 (+),EMA (+),S100 (−),Desmin (−),SMA (−)	Deep femoral artery, Superficial femoral artery	Superficial femoral artery and vein, Deep femoral artery, Superficial femoral artery, Great saphenous vein, Femoral nerve, Adductor brevis, Adductor longus, vastus medialis, sartorius
1	2023	(3)	F	13	Anteromedial aspect of right thigh	10 × 5	Biphasic	N	N	N	N/A	MIB-1 (+),CD99 (+),TLE (+)	N/A	Superficial femoral artery and vein
2	2023	(5)	M	59	Lower end of left thigh	4 × 4	Monophasic	N/A	N	N/A	SS18	pan-cytokeratin (+),S100 (+),SOX10 (−),actin (−),CD34 (−),desmin (−)	N/A	N/A
3	2022	(3)	F	29	Right thigh	N/A	Monophasic	N/A	Y	N/A	N/A	N/A	N/A	N/A
4	2022	(6)	F	16	Proximal part of the left thigh	6.4 × 4.8	PD	N/A	Lung, lumbar spine (10 Months)	Died (14 months)	SS18	CD99 (+),CD56 (+),Bcl-2 (+),TLE1 (+),S100 (−),NSE(−),NKX2.2 (−),TTF-1 (−),Myogenin (−),MyoD1 (−),LCA (−),SATB2 (−),Syn (−),CgA(−),CK(−),SMA (−),Desmin (−)	N/A	N/A
5	2021	(7)	M	68	Right thigh	5 × 4	N/A	Y (5 months)	Right Lung, Left Atrial, Right Adrenal Gland	N/A	ss18	SDHB(+),CgA(−),Syn (−),ALK-1 (−),MART-1 (−),CR (−),S-100 (−),HMB45(−),Cytokeratin (−),EMA (−),CD34 (−),CD31 (−),SMA (−)	N/A	N/A
6	2021	(30)	F	46	Distal dorsal side of the left thigh	7	Monophasic	N	N	N	SS18/SSX	N/A	N/A	Sciatic nerve
7	2021	(12)	M	60	Left thigh	12	Biphasic	N/A	Lung	N/A	SS18	N/A	N/A	Femoral nerve, Femoral vein
8	2020	(7)	M	30	Left thigh	3	Monophasic	Y (6 months)	Pancreas (15 Months)	Died (27 months)	N/A	bcl2 (+),Cytokeratin (+),EMA (+),CK(+),CgA(−),SYP(−),Vimentin (−)	N/A	N/A
9	2020	(4)	F	25	Left posterior and medial thigh	9.8 × 10×11.8	N/A	N/A	N/A	N/A	N/A	TLE-1 (+)	N/A	N/A
10	2020	(2)	F	22	Left thigh	8.0 × 8.4×10.1	Monophasic	N/A	N/A	N/A	t (X; 18)	vimentin (+),CD56 (+),TLE1 (+)	N/A	Adductor group of muscles
11	2019	(4)	F	28	Left thigh	14 × 10×8	N/A	N/A	N/A	N	N/A	N/A	N/A	Adductor longus muscle, Sartorius muscle
12	2019	(4)	M	35	Medial aspect of the right thigh	10	Biphasic	N/A	N/A	N/A	N/A	pan CK(+),CK7(+),CK19(+),EMA (+),bcl2 (+)	N/A	Rectus femoris muscle
13	2019	(4)	F	18	Right thigh	N/A	N/A	N/A	N	N/A	N/A	N/A	N/A	Sciatic nerve, Bicep femoris
14	2019	(10)	F	39	Left thigh	8	N/A	Y (14 months)	Lung (10 months)	N/A	N/A	N/A	N/A	N/A
15	2017	(3)	M	24	Left thigh	4.4 × 2.1	N/A	N/A	N/A	N	N/A	N/A	N/A	N/A
16	2017	(2)	M	42	Medial of his right thigh	4.1 × 4.3×4.6	Monophasic	N/A	N/A	N/A	SYT-SSX2	EMK(+),Cytokeratin (+),actin (−),desmin (−),S100 (−),CD31 (−),CD34 (−),CD99 (−)	N/A	Femoral vein
17	2016	(1)	M	56	Left upper thigh	9.5	N/A	N/A	Lung, Focal mucosal of the stomach	N/A	N/A	Vimentin (+),Cytokeratin (+),EMA (+),BCL2(+),KIT (−),CD3 (−)4,CD99 (−),Desmin (−),DOG 1 (−),SMA (−)	N/A	N/A
18	2016	(20)	M	32	Left pelvis and femur		Monophasic	N	Pancreas	N	SS18-SSX1 or SS18-SSX2 chimera	bcl-2 (+),pan-cytokeratin (−),EMA (−),chromogranin A (−),synaptophysin (−),vimentin (−)	N/A	N/A
19	2015	(6)	F	19	Left upper thigh	13 × 15	Biphasic	N	N	N	t (X; 18) (p11; q11)	bcl (+),EMA (+),CD34 (+)	Deep femoral artery	Sciatic nerve, Deep femoral artery
20	2015	(5)	M	17	Right thigh	N/A	N/A	N/A	Chest wall, Lung	N/A	N/A	N/A	N/A	N/A
21	2012	(3)	F	65	Medial compartment of the thigh	99 × 78×73	N/A	N/A	N	N/A	N/A	N/A	N/A	N/A
22	2012	(5)	M	57	Proximal part of the right femur	6.5 × 6.0×6.0	Biphasic	N/A	N/A	Died	N/A	vimentin (+),cytokeratin 7 (+),EMA (+),pan-cytokeratin (+)	N/A	N/A
23	2012	(4)	M	75	Distal shaft of femur	12 × 10×8	Monophasic	N/A	Lung	N/A	N/A	vimentin (+),EMA (+),BCL2(+),S100 (−),Desmin (−),Actins (−),CD99 (−),CD45 (−),CAM 5.2 (−),AE13 (−)	N/A	N/A
24	2009	(3)	M	44	Right biceps femoris muscle	N/A	N/A	N/A	N/A	N/A	N/A	N/A	N/A	Sciatic nerve, Biceps femoris muscle
25	2006	(6)	M	36	Anterior aspect of the right thigh	18 × 10×11	N/A	N/A	Lung	N/A	N/A	N/A	N/A	Vastus intermedius, Vastus medialis muscles

M, male; F, female; PD, poorly differentiated; N/A, not available; SDHB, dehydrogenase complex iron sulfur subunit B.

Of this literature, only 6 explicitly reported recurrence of the original disease. Among them, the recurrence occurred in 2 cases, and the other 4 cases recurrence did not occur. Distant metastasis was explicitly reported in 17 articles. Of these, 11 patients developed metastases, while the other 6 did not. Of the cases in which metastasis occurred, 6 metastasized to lung tissue, 2 to the pancreas, and one each to the chest wall, endocardium, adrenal glands, stomach wall, and lumbar spine. Nine reports clearly documented the deaths of patients. Of these reports, three patients died within 5 years of lesion detection, whereas the other six patients did not experience a fatal event during the follow-up period. In the literature, postoperative follow-up for patients with SS primarily relies on imaging examinations, focusing on chest CT, abdominal ultrasound, bilateral lower limb MRI, and PET-CT to detect subtle metastatic lesions that may be challenging to identify with different imaging modalities.

Out of these 25 case reports, only 1 paper mentioned that the lesion was supplied by the deep femoral artery. In this case, radiographic examination showed that the blood supply to the lesion came from branches of the superficial femoral artery and the deep femoral artery. In 11 of these reports, the tissues involved in the lesion were described, including the sciatic nerve in 4 cases, the femoral nerve in 1 case, the femoral vein in 2 cases, the superficial femoral artery in 1 case, the superficial femoral vein in 1 case, the biceps femoris muscle in 2 cases, the rectus femoris muscle in 1 case, the middle femoral muscle in 1 case, the adductor femoris muscle in 1 case, and the adductor longus muscle in 1 case. For this case, we concluded from the imaging presentation and intraoperative situation that the lesion invaded several arteries, such as the right superficial femoral artery and the deep femoral artery, and veins, such as the superficial femoral vein and the great saphenous vein. There was also invasion of the femoral nerve, as well as several muscle tissues, including the adductor brevis, adductor longus, part of vastus medialis and sartorius.

Imaging examination plays an important role in the localization and characterization of the disease, and provides guidance for the treatment of the disease. Of these 25 case reports, only 4 described the ultrasonographic appearance of the lesion (Table 2). These reports showed a mixed echogenic mass of the lesion, in which calcification was seen in 1 case, areas of echo-less liquefaction were 2 in two cases, and a small amount of blood flow signal was seen in the lesion on color Doppler. In contrast, the ultrasound presentation of the lesion in this case was a slightly hypoechoic mass, with no clear areas of calcification or liquefaction observed. In addition, the lesion and the adjacent superficial femoral vein and great saphenous vein were not clearly visualized, while the blood flow signal in the lesion was slightly rich on color Doppler. Based on this case, we can summarize that the SS is a solid hypoechoic mass located near the joint, with variable size, well-defined borders, and no invasion of adjacent joints. The smaller masses have relatively regular shapes and homogeneous internal echogenicity, while the larger ones tend to have irregular shapes and heterogeneous internal echogenicity, with possible occurrences of liquefaction and calcification. Color Doppler imaging reveals abundant blood flow signals within the mass, exhibiting a patchy or branching distribution.

**TABLE 2:** Reported the imaging findings of primary synovial sarcoma of thigh.

Case no.	Year	Author	Ultrasound	MRI
	2023	Our case	A hypoechoic mass with a size of 7.2 × 5.6 × 6.4 cm was observed in the vastus medialis muscle of the middle and lower part of the right thigh, with irregular shape, unclear boundary and uneven internal echo. The superficial femoral vein and great saphenous vein around the mass were not clearly displayed. Color Doppler imaging showed rich blood flow signals in the mass	A lobulated soft tissue mass in the middle of the right thigh (centered on the adductor magnus and adductor longus, and part of the vastus medialis), with slightly high signal intensity on T1WI and T2WI, and extensive diffusion restriction on DWI sequence. The mass was well demarcated from the right femur, with intact cortex and no abnormal signal lesions in the medullary cavity
1	2023	(3)	N/A	N/A
2	2023	(5)	N/A	A multilobulated irregular mass located just deep to the medial patellofemoral retinaculum and inferior to the vastus medialis muscle. The mass demonstrated slight hyperintense T1 signal relative to muscle and heterogeneous, predominantly hyperintense T2 signal with enhancement on the post-contrast sequences
3	2022	(3)	N/A	N/A
4	2022	(6)	N/A	A bone tumor in the proximal part of the left femur. The mass showed isointensity on T1-weighted images and high intensity on T2-weighted images (T2WI) and was heterogeneously enhanced by gadolinium-diethylenetriamine pentaacetic acid
5	2021	(7)	N/A	N/A
6	2021	(30)	N/A	A well-circumscribed 7 cm lobulated soft tissue tumor with cystic change on the proximal side of the femur
7	2021	(12)	N/A	A mass adjacent to the femoral nerve and the femoral vein in the patient’s left thigh, with a low intensity in T1-weighted image and mixed intensity of high and low in T2-weighted image
8	2020	(7)	N/A	N/A
9	2020	(4)	N/A	A large multilobulated solid lesion in the intra- and intermuscular plane of adductor muscles and obturator externus T1W isointense,T2W,and STIR hyperintense
10	2020	(2)	A heteroechoic space occupying lesion in the upper part of left thigh, mainly involving the adductor group of muscles	
11	2019	(4)	N/A	A large heterogeneous soft tissue mass within the left medial thigh, which had replaced most of the distal fibres of the left adductor longus muscle belly, was encasing the femoral vessels. Infiltration of the left sartorius muscle was also noted in that region with no signs of osseous infiltration
12	2019	(4)	N/A	A large lobulated hypervascular mass lesion in the deep portion of the right rectus femoris muscle
13	2019	(4)	N/A	N/A
14	2019	(10)	N/A	N/A
15	2017	(3)	An oval, heterogeneous structure containing calcifications adjacent to a hypoechoic region. A focal area of vascularity was seen on color Doppler imaging between the calcifications and hypoechoic area. Sonographic view in long axis showed an eccentrically positioned mass with proximal and distal areas suggestive of tapering thought to initially be an entering and exiting nerve	The left thigh with and without contrast was obtained and revealed a lobulated, anterolateral vastus lateralis sub-facial mass with uniform enhancement and areas of calcification
16	2017	(2)	N/A	N/A
17	2016	(1)	N/A	A large heterogeneous left upper thigh mass with malignant features (capsular breach and intramuscular extension)
18	2016	(20)	The lesion was depicted as a well-demarcated and heterogeneous mass with abundant cystic components. Although the mass showed expansive growth and oppressed the splenic vein,no invasion was suspected	The mass was depicted as a hypo-, hyper-, and hyper-intense lesion on T1-,T2-,and diffusion-weighted images, respectively. It was heterogeneously enhanced on arterial-phase images
19	2015	(6)	N/A	N/A
20	2015	(5)	N/A	N/A
21	2012	(3)	N/A	A large soft tissue tumour located in the medial compartment. The tumour was inhomogeneous without visible capsule and was surrounded by compressed tissues. Visible vascular modelling
22	2012	(5)	N/A	An expansile lesion at the proximal femur in the intertrochanteric region
23	2012	(4)	N/A	A large soft tissue mass around the right distal shaft of femur, with a large zone of transition and associated cortical destruction
24	2009	(3)	N/A	N/A
25	2006	(6)	A large heterogeneous mass of mixed echotexture with a central area of liquefaction consistent with an intramuscular haematoma	T1 weighting (T1W) demonstrated a lesion with peripheral high signal. Gradient-echo T2-weighted images revealed marked signal drop out centrally due to magnetic susceptibility artefact from altered blood products, usually haemosiderin. The mass was shown to extend posteriorly as far as the medial cortex of the femur and medially to the femoral canal

N/A, not available.

Ultrasound can demonstrate the extent of involvement of surrounding muscles and blood vessels, as well as the vascular supply; however, a definitive diagnosis of SS still requires differentiation from other diseases, such as malignant peripheral nerve sheath tumor, malignant fibrous histiocytoma, invasive fibroma, etc. Malignant peripheral nerve sheath tumors are commonly found in the thigh, buttock, upper arm, and paravertebral region. They typically present as heterogeneous hypoechoic masses, which may show irregular, varying thickness strands within. Cystic changes may be observed inside, and the margins may exhibit a capsule-like strong echogenicity. Malignant fibrous histiocytoma typically runs parallel to the long axis of the body and is located within the muscle layer or subcutaneously, which is a key distinguishing feature. Invasive fibroma presents as a hypoechoic mass with interspersed areas of hyperechogenicity. Expansile growths appear as rounded or oval shapes, with some showing a pseudocapsule.

MRI has good soft tissue resolution, especially in the nervous system and bone and joint systems. Of these 25 case reports, 13 described the MRI manifestations of the lesions (Table 2). MRI showed the lesion to be a soft tissue signal with iso-or hypointensity on T1-weighted images and mixed high and low signal on T2-weighted images, and enhancement scans showed the lesion to show inhomogeneous enhancement. Only one of these reports described a lesion with invasion of adjacent muscles and encircling the femoral artery. “Triple-sign” is always mentioned in the literature (30), that is, synovial sarcoma has three signal areas of high, medium and low signal in T2-weighted images. In this case, MRI showed isointense and hyperintense T2-weighted images of the lesions, hyperintense signals of cystic degeneration and small pieces of necrosis, and hypointense signals of calcification, fibrous components and old hemorrhage. The lesion invaded the adductor brevis, adductor longus and part of the adductor femoris, but did not invade the right femur. The lesion wrapped around the right superficial femoral artery, indicating the difficulty of surgery. When the fascia of the muscle tissue surrounding the lesion is unclear and there is a disruption in the normal alignment of the muscle fibers, the possibility of invasion should be considered. When the lesion encases a blood vessel or is located adjacent to one, with the blood vessel appearing tortuous and enlarged, the possibility of invasion should be considered.

CT is not the preferred imaging modality for soft tissue lesions, as evidenced by the reported cases and this case, which did not include CT scans of the primary lesion. However, the study by Mickael et al. indicates that CT is more sensitive to calcific components within the lesion (30). The presence of calcification in SS lesions is associated with better overall survival, and CT imaging can be helpful in predicting the prognosis of SS patients. Ultrasound examination can partially reveal the presence of hemorrhage, necrosis, and calcification within SS lesions, show whether the lesion has a capsule, and provide a more visual assessment of blood flow in the lesion’s center, as well as its proximity and relationship with surrounding blood vessels. MRI can perform multiparametric high-contrast imaging of SS lesions, providing more comprehensive diagnostic information. This enhances the ability to characterize the lesions in detail, assess their extent, and evaluate their relationship with adjacent structures. However, MRI is not sensitive to the detection of calcification within lesions and the surrounding cortical bone. This limitation can affect the assessment of certain characteristics of the lesions. CT and PET scans are better suited for detecting distant metastases, providing valuable information about the presence and extent of metastatic disease. Therefore, the diagnosis of SS should integrate multiple imaging modalities to obtain a comprehensive assessment of the lesions and their characteristics.

Among the 25 case reports, 15 mentioned pathological classification, of which 9 cases were monophasic type, 5 cases were biphasic type, and only 1 case was undifferentiated type. In view of this case, the microscopic observation showed that the epithelioid tumor cells and spindle tumor cells were mixed and densely proliferated, presenting nested clusters and striated distributions, with characteristics of infiltrative growth, which was in line with the manifestation of biphasic tumors. SS18 gene translocation was detected in 9 cases. Immunohistochemical testing was performed in 13 cases, of which 5 were positive for Bcl-2 antibodies and 7 were positive for EMA. For this patient, immunohistochemistry results showed positive Bcl-2 antibodies and EMA, and these results helped to confirm the diagnosis of SS.

## Conclusion

In conclusion, synovial sarcoma is a rare malignant mesenchymal tumor, so its accurate diagnosis is essential for treatment and prognosis. We report the diagnosis of biphasic synovial sarcoma in a 16-year-old Chinese female, and we provide a comprehensive and detailed description of her clinical symptoms, imaging manifestations, surgery, and pathologic findings. Synovial sarcoma in the imaging can show certain characteristics, but its specificity is low, need to identify with a variety of other soft tissue tumors. Histopathological and immunohistochemical findings are still needed to make a definitive diagnosis.
